# Smoking is a predominant risk factor for coronary artery disease among Indians

**DOI:** 10.6026/973206300200719

**Published:** 2024-07-31

**Authors:** Rafae Taqiuddin, Mohammed Jaffer Ali, Aishwarya Kimmatkar, Nimerta Lohana, Anveshak, Maarya Mohammed Siddiqui, Yasir Adil El Rashid Mohamed, Mohamed Elfatih Musaab Ibrahim Mohamed, Rida Minhaj, Mohammed Abdul Mateen

**Affiliations:** 1 Shadan Institute of Medical Sciences, Teaching hospital and Research Centre, Hyderabad, India; 2 Bhaskar Medical college and Bhaskar General Hospital, Hyderabad, India; 3 International School of Capital Medical University, Beijing, China; 4 Liaquat University of medical and Health Sciences, Jamshoro, Sindh, Pakistan; 5 Hassan Institute of Medical Sciences, Karnataka, India; 6 Shadan Institute of Medical Sciences, Teaching Hospital and Research Centre, Hyderabad, India; 7 Military Medical Hospital, Omdurman, Khartoum, Sudan; 8 National Ribat University, Khartoum, Sudan; 9 Deccan College of Medical Sciences, Hyderabad, India; 10 Shadan Institute of Medical Sciences teaching hospital and Research Centre, Hyderabad, India

**Keywords:** Smoking, coronary artery disease, Indians, risk factor, public health, tobacco control

## Abstract

Smoking has emerged as a predominant risk factor for coronary artery disease (CAD) in India, contributing significantly to the country's rising cardiovascular morbidity and mortality rates. The adverse effects of tobacco on cardiovascular health are well-documented, exacerbating a public health challenge in a nation with a high prevalence of smoking. Therefore, it is of interest to analyse the impact of smoking on the incidence and progression of coronary artery disease among the Indian population, emphasizing the need for targeted public health interventions to mitigate this risk factor. We included 1000 adults were enrolled from January to July 2023. The group comprised 500 CAD patients (cases) and 500 individuals without CAD (controls). We gathered information on demographics, smoking habits & other CAD risk factors. To assess the relationships between smoking, CAD, and other variables, we utilized multivariate logistic regression. The analysis revealed that current smokers had a substantially increased likelihood of CAD, with an unadjusted odds ratio (OR) of 3.20 (95% CI: 2.45-4.18), compared to non-smokers. This association remained significant even after adjusting for confounders, with an adjusted OR of 2.80 (95% CI: 2.10-3.75). The study confirms smoking as a critical, Adaptable risk element for CAD, independently increasing the risk of the disease. Smoking significantly elevates the risk of developing coronary artery disease among Indians. Comprehensive anti-smoking campaigns and stringent tobacco control policies are imperative to reduce the burden of CAD. Public health strategies must focus on awareness, prevention, and cessation support to combat this major health threat effectively.

## Background:

Coronary Artery Disease (CAD) continues to be a predominant health issue globally, contributing significantly to both morbidity and mortality. The medical sectors pay particular attention to CAD, as it remains a main cause of mortality and an important concern in the realm of cardiac health. A thorough comprehension of the risk factors connected to CAD is very crucial for developing effective prevention and treatment strategies. Among the various factors contributing to CAD's complex etiology, cigarette smoking is identified as a key controllable risk factor. A substantial body of epidemiological and clinical studies has established a strong association between smoking and the development, progression, and severity of CAD [1, 2]. As per World Health Organization (WHO), as of 2018, the global population of smokers stood at around 1.3 billion, predominantly due to tobacco use [3]. The habit of smoking is influenced by a broad spectrum of social, environmental, and individual factors. Despite the overwhelming evidence linking smoking to various health risks, it remains a prevalent practice in many communities, with significant cardiovascular implications, particularly concerning the coronary arteries. Extensively supports the notion that smoking accelerates the atherosclerotic process, which is the primary pathological basis for CAD [4, 5]. The harmful constituents of cigarette smoke, such as nicotine and carbon monoxide, have deleterious effects on the cardiovascular system. Smoking contributes to the initiation and progression of atherosclerosis through mechanisms involving endothelial dysfunction, oxidative stress, inflammation, and pro-thrombotic states [6]. Additionally, the impact of smoking on CAD extends beyond atherosclerosis. There is a connection between smoking and increased cardiovascular mortality, a higher risk of myocardial infarction (MI), and more severe coronary lesions [7, 8]. It also adversely affects outcomes in CAD patients undergoing revascularization techniques such as PCI and coronary artery bypass grafting (CABG) [9-10]. Therefore, it is of interest to explore the specific pathways through which smoking affects the coronary arteries and the extent of its risk contribution.

## Material and Methods:

## Research design:

This research employed a case-control approach to explore the link between smoking and coronary artery disease (CAD). By adopting this methodology, the study aimed to compare the smoking habits and other pertinent variables of individuals diagnosed with CAD (the cases) to those without the disease (the controls).

## Participant demographics:

Adults aged 18 to 75 were recruited for this study from January to July 2023. Those diagnosed with CAD, as confirmed by methods like coronary angiography showing significant stenosis, positive stress tests, or cardiac catheterization findings, were categorized as cases. Controls, selected from the same demographic pool, had no history of CAD, verified via medical records and physical examinations.

## Data collection process:

Data was collected through a combination of structured interviews and examination of medical records. Participants underwent interviews with trained personnel using standard questionnaires to gather details about their demographics, smoking patterns. Clinical data regarding CAD diagnosis and severity were extracted from electronic health records, with smoking status verified through both medical documentation & biochemical markers such as cotinine levels.

## Evaluation of smoking exposure:

Current smokers were those who smoked regularly up to the past 6 months, while former smokers had ceased smoking at least 6 months prior to participating. Never-smokers were individuals who had never engaged in regular cigarette smoking.

## Consideration of confounding variables:

The study considered various confounders like age, body mass index (BMI), gender, diabetes, hypertension, lipid levels, and other known CAD risk factors based on existing literature.

## Statistical analysis:

Epi Info version 7 software was utilized for statistical analysis. The mean, standard deviation, frequencies, and percentages of descriptive statistics were employed to delineate the clinical and demographic features of the case and control groups. Bivariate analyses (chi-square tests, t-tests) assessed the relationship between smoking status & CAD. Then, using multivariate logistic regression, the odds ratios (ORs) and 95% confidence intervals (CIs) for the relationship between smoking and CAD were determined, adjusting for potential confounders.

## Ethical Consideration:

The study complied with the Declaration of Helsinki's ethical standards. Before any subject was included in the study, their informed permission was acquired and ethical approval was received from the appropriate institutional review board.

## Results:

Table 1 presents the findings of participants, encompassing both CAD patients (cases) and those without CAD (controls). The average age for the case group was found to be 63.5 years, with a standard deviation (SD) of 8.2, while the control group had an average age of 62.0 years, with an SD of 7.5, indicating comparable age distributions between the groups. In the case group, males constituted 64%, & in the control group, 66% were male, suggesting a similar gender ratio in both categories. The mean BMI was observed to be marginally higher in the case group (28.6 kg/m^2^; SD 4.3) as opposed to the control group (27.3 kg/m^2^; SD 3.9). Regarding hypertension, a larger proportion of the case group (76%) experienced it compared to the control group (44%). Diabetes was more prevalent among the case group (48%) in contrast to the control group (32%). Additionally, a family history of CAD was reported more frequently in the case group (36%) than in the control group (24%).

Table 2: The study categorizes participants into current smokers, former smokers, and never smokers, highlighting median pack-years as an index of smoking exposure. Among the CAD group (cases), 44% were current smokers, compared to 20% in the non-CAD group (controls). Former smokers made up 36% of the case group and 48% of the control group, while never smokers were 20% of the case group and 32% of the control group. The median pack-years for current smokers in the case group was 25 (IQR 15-35), indicating a significant smoking history, whereas the control group's current smokers had a lower median pack-years at 10 (IQR 5-20). Table 3: The severity of CAD among the case group is categorized into mild, moderate, and severe. Mild CAD was observed in 20% of cases, moderate CAD in 50%, and severe CAD in 30%. This table provides a clear distribution of CAD severity within the diagnosed participants. Table 4: This table presents the odds ratios (ORs) for developing CAD based on smoking status, both unadjusted and adjusted for confounding variables. The unadjusted OR for current smokers developing CAD was 3.20 (95% CI 2.45-4.18). After adjusting for factors like age, gender, hypertension, and diabetes, the OR for current smokers slightly decreased to 2.80 (95% CI 2.10-3.75), still indicating a strong link between current smoking and increased CAD risk. Table 5: The table shows adjusted odds ratios (ORs) for various factors, considering their influence on CAD when other variables are included in the analysis. Each decade increase in age corresponds to a 25% higher likelihood of developing CAD (adjusted OR 1.25, 95% CI 1.10-1.43). Male gender is also significantly associated with a higher risk of CAD, with an adjusted OR of 1.80 (95% CI 1.50-2.15), highlighting the impact of these factors on CAD risk.

## Discussion:

The findings from this case-control study shed light on the relationship between smoking and coronary artery disease (CAD), uncovering key insights about the impact of smoking status, CAD risk, & the role of various confounding factors. Analysis supports the established association between smoking and an increased risk of CAD. Current smokers were found to have a notably higher unadjusted odds ratio (OR) of 3.20 (95% CI 2.45-4.18) compared to never smokers, affirming the detrimental impact of active smoking on cardiovascular health. This aligns with the extensive exploration in this area, underlining the critical importance of smoking cessation programs in CAD prevention [5,7, 11]. Adjusting for confounders like age, gender, diabetes, hypertension & family history of CAD, the association between current smoking & CAD remained significant, with an adjusted OR of 2.80 (95% CI 2.10-3.75). This underscores the significant & independent effect of smoking on CAD risk, lending support to the complex mechanisms through which smoking contributes to atherosclerosis and CAD [4, 6, 7, 11]. This study also reveals that former smokers, while at a lower risk compared to current smokers, still face an elevated risk of CAD compared to never smokers. The unadjusted OR for former smokers was 1.60 (95% CI 1.25-2.05), indicating the lasting effects of past smoking habits [7, 8]. The relationship between CAD severity and smoking was also examined. Both current and former smokers showed higher incidences of moderate and severe CAD, yet because there may have been bias in the case group's selection, these results should be regarded cautiously. Further studies with larger samples and more comprehensive evaluations of CAD severity are needed to elucidate this relationship. In this study multivariate analysis, other significant risk factors for CAD were identified. Age showed a clear dose-response relationship with CAD risk, increasing by 25% with every decade [1]. Male gender emerged as a significant predictor, nearly doubling the risk compared to females [2]. Additionally, diabetes & hypertension were independently associated with increased CAD risk, emphasizing the importance of managing these conditions in CAD prevention strategies [10]. A strong family history of CAD also appeared as a significant risk factor, highlighting genetic predispositions [9].

## Conclusion:

Data shows that smoking as a key risk factor for CAD among Indians. Hence, there is a need for tobacco use reduction and smoking cessation initiatives to decrease CAD burden. Future studies are needed on clarifying the dose-response relationship, evaluating smoking cessation interventions, & exploring novel preventive strategies for CAD.

## Limitations:

Limitations of study include the observational nature, potential recall bias in self-reported smoking history and selection bias in the case group. The dose-response relationship between smoking intensity & CAD risk warrants further investigation.

## Figures and Tables

**Table 1:** Demographic Characteristics of Study Participants

**Characteristic**	**Cases (n=500)**	**Controls (n=500)**
Age (years), mean (SD)	63.5 (8.2)	62.0 (7.5)
Gender (Male/Female), n (%)	320 (64%)	330 (66%)
BMI (kg/m^2^), mean (SD)	28.6 (4.3)	27.3 (3.9)
Hypertension, n (%)	380 (76%)	220 (44%)
Diabetes, n (%)	240 (48%)	160 (32%)
Family History of CAD, n (%)	180 (36%)	120 (24%)

**Table 2:** Smoking Status Among Study Participants

**Smoking Status**	**Cases (n=500)**	**Controls (n=500)**
Current Smokers	220 (44%)	100 (20%)
Former Smokers	180 (36%)	240 (48%)
Never Smokers	100 (20%)	160 (32%)
Pack-Years, median (IQR)	25 (15-35)	10 (5-20)

**Table 3:** Severity of coronary artery disease among cases

**Severity Grade**	**Number of Cases (%)**
Mild	100 (20%)
Moderate	250 (50%)
Severe	150 (30%)

**Table 4:** Odds Ratios (ORs) for CAD risk associated with smoking

**Smoking Status**	**Unadjusted OR (95% CI)**	**Adjusted OR (95% CI)**
Current Smokers	3.20 (2.45-4.18)	2.80 (2.10-3.75)
Former Smokers	1.60 (1.25-2.05)	1.45 (1.10-1.92)
Never Smokers	Reference	Reference

**Table 5:** Multivariate Analysis of CAD Risk Factors

**Risk Factor**	**Adjusted OR (95% CI)**
Age (per 10 years)	1.25 (1.10-1.43)
Male Gender	1.80 (1.50-2.15)
Hypertension	2.10 (1.75-2.55)
Diabetes	1.40 (1.15-1.70)
Current Smoking	2.80 (2.10-3.75)
Family History of CAD	1.60 (1.30-1.95)
